# The Antiseptic Properties of Saliva

**Published:** 1897-04

**Authors:** 


### The Antiseptic Properties of Saliva.

Hugenschmidt (*Ann de l' Inst. Pasteur*, October, 1896) has made an attempt to determine why operations upon the mouth are so seldom followed by infection. He was not able to prove that the saliva possesses any germicidal power. The more common bacteria grow rapidly in it. Nevertheless, it has a two-fold mechanical action to keep down infection. It dilutes and washes away alimentary material, and by its alkalinity it prevents fermentation.

But Hugenschmidt thinks the saliva possesses the power of stimulating diapedesis in the numerous lymphatic organs situated in the mouth. Even in its normal state the saliva contains products of the secretion of microbes, and therefore possesses an attraction for microbes, and thus favors the phagocytic action.

This hypothesis the writer upholds by several experiments. One of them was the introduction of small glass tubes into the abdomen of the guinea-pig. These tubes were closed at one end and filled with saliva In eight hours it was found that the leucocytes had crowded into the open end of the tube, forming a plug about two mm. in thickness.

In a second experiment, a small portion of the mucous membrane of the lower jaw of the guinea-pig was resected and its base mangled with a curette. In twenty-four hours the wound was covered with a layer of leucocytes, in the interior of which were found bacteria of various sorts, apparently of the same sorts as those in the saliva. From these, and other experiments, Hugenschmidt concludes that the saliva has no germicidal power, and that the freedom from infection of wounds in the mouth is due to active phagocytosis, brought about about by the power of the saliva to stimulate the diapedesis of leucocytes.—*Med. News.*

---

THE *Johns Hopkins' Hospital Bulletin* advocates the use of absolute alcohol as a disinfectant, and for all cutting instruments used in operations upon the eye. It is the best substitute for heat, which latter, both moist and dry, dulls the edge of instruments.

